# Carbamate-directed benzylic lithiation for the diastereo- and enantioselective synthesis of diaryl ether atropisomers

**DOI:** 10.3762/bjoc.7.156

**Published:** 2011-09-26

**Authors:** Abigail Page, Jonathan Clayden

**Affiliations:** 1 School of Chemistry, University of Manchester, Oxford Rd., Manchester M13 9PL, UK

**Keywords:** configurational stability, diaryl ether, diastereoselective, enantioselective, lateral lithiation, metallation

## Abstract

Diaryl ethers carrying carbamoyloxymethyl groups may be desymmetrised enantio- and diastereoselectively by the use of the *sec*-BuLi–(−)-sparteine complex in diethyl ether. Enantioselective deprotonation of one of the two benzylic positions leads to atropisomeric products with ca. 80:20 e.r.; an electrophilic quench typically provides functionalised atropisomeric diastereoisomers in up to 97:3 d.r.

## Introduction

**Scheme 1:**
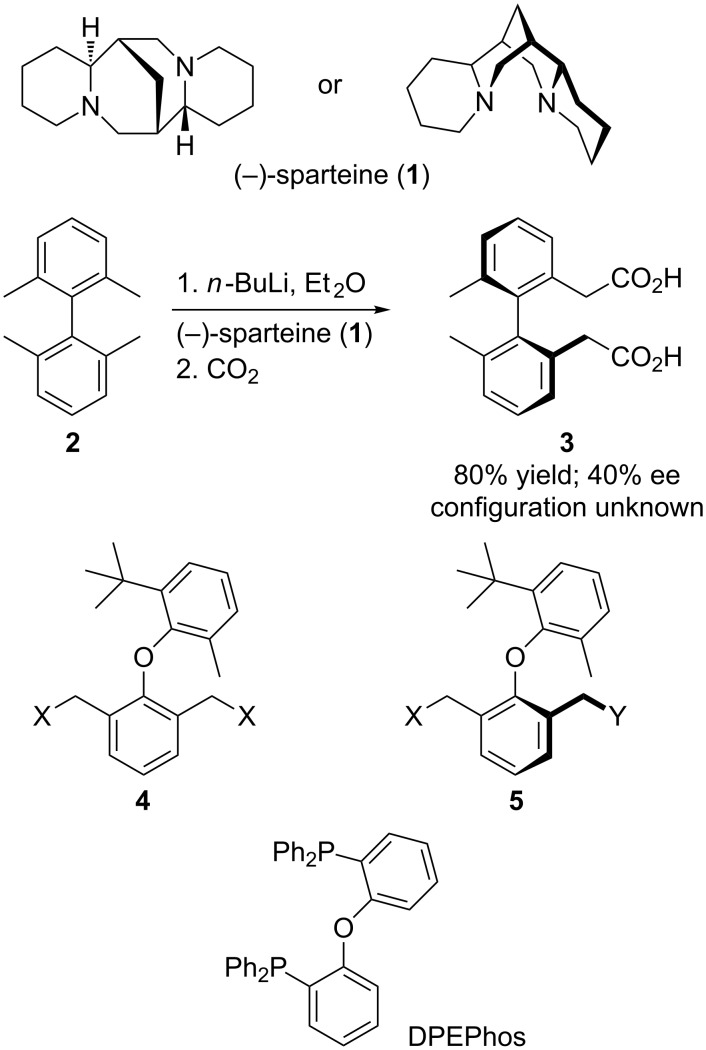
Desymmetrising metallation for the enantioselective synthesis of atropisomers.

One of the first uses of the chiral diamine (−)-sparteine (**1**) in its now familiar role of controlling asymmetric deprotonation/electrophilic quench reactions [1] was an enantioselective benzylic deprotonation of bis(2,6-dimethylbenzene) with the aim of generating an atropisomeric product in enantiomerically enriched form (Scheme 1) [2]. Enantioselective lithiation has since then commonly been used to generate axial or planar chirality [3], in many cases by desymmetrising deprotonation of a functionalised aromatic system. The relative acidity of benzylic protons makes enantioselective deprotonation in the style of Scheme 1, of one of a pair of enantiotopic aromatic methyl, alkoxymethyl or acyloxymethyl substitutents, a viable approach to the asymmetric synthesis of atropisomeric molecules.

We have developed a number of methods for the synthesis of “nonstandard” atropisomer structures containing rigid fragments joined by a hindered single bond, but which are different from the typically well-studied biaryl compounds [4–5]. These non-biaryl atropisomers have included aromatic amides [6–8], ureas [9], ethers [10–12], sulfides and sulfones [13], and many of the methods we developed for their asymmetric synthesis employed dynamic resolution techniques under kinetic or thermodynamic control [6,11,13–16]. The mechanistic possibilities associated with dynamic resolution were initially elaborated by Beak for organolithium compounds having varying degrees of configurational stability [17–18], and in our studies on ureas and amides we were able to identify correlated inversion processes linking configurational inversion at organolithium centres with conformational inversion of atropisomeric chirality by bond rotation [19]. Several of the classes of atropisomers we have studied contain functional groups which are excellent directors of lithiation [20], and indeed our original interest in lithiation stemmed from the need to build these atropisomeric structures rapidly and efficiently [21].

We recently reported on the enantioselective synthesis of diaryl ethers (a potential new class of chiral ligand having a structure related to the wide bite angle diphosphine DPEPhos) by biocatalytic oxidation or reduction, and which made use of desymmetrisation of a “pro-atropisomeric” substrate **4** to achieve the required enantiomeric enrichment in the product **5** [22]. In this paper we report parallel studies on the attempted asymmetric synthesis of atropisomeric diaryl ethers in diastereomerically and/or enantiomerically enriched form by the directed deprotonation and electrophilic quench of benzylic positions ortho to a sterically hindered diaryl ether linkage.

## Results

**Scheme 2:**
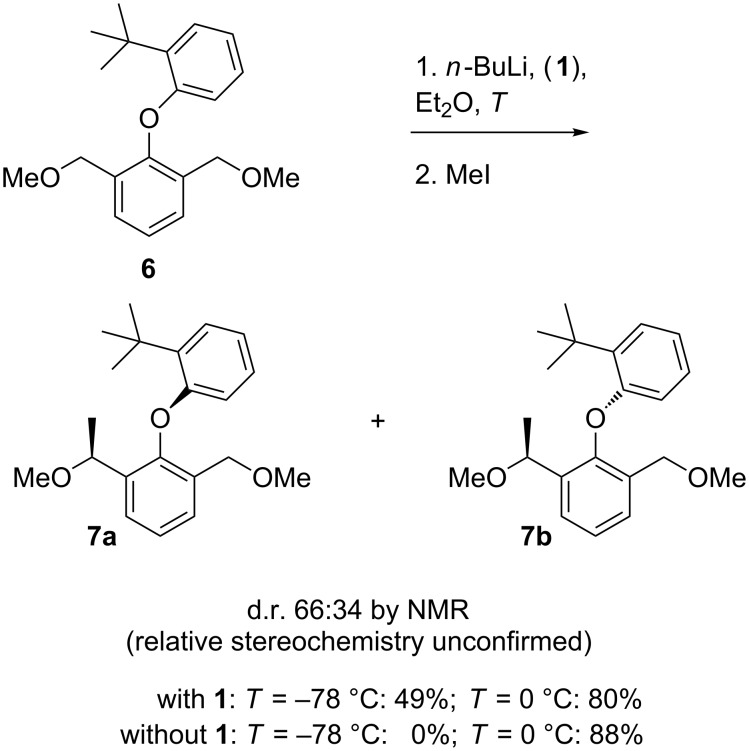
Benzylic lithiation of a diaryl ether.

An aryloxy group is a weak director of metallation [20,23], but in preliminary studies we were able to deprotonate and methylate the hindered diaryl ether **6** [10] by treatment with *n*-BuLi in ether at 0 °C with or without (−)-sparteine (Scheme 2). Methylation of the resulting organolithium returned the product **7** in up to 88% yield as a mixture of diastereoisomers (by NMR). Previous data on the conformational stability of related diaryl ether [10], coupled with our inability to separate these diastereoisomers, and the invariant ratio in which they were obtained, suggested that they are insufficiently hindered to exist as stable atropisomers and they interconvert on a relatively short timescale, of seconds to minutes, at room temperature.

**Scheme 3:**
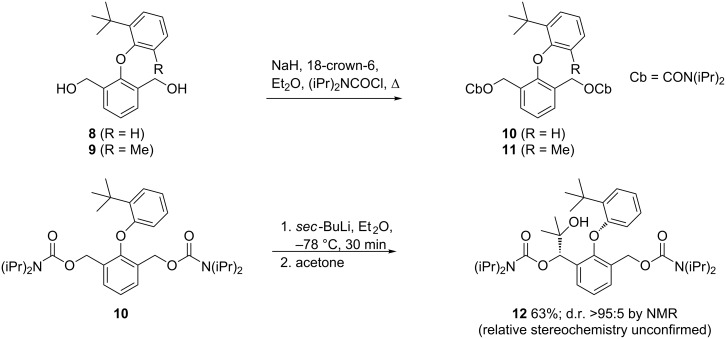
Benzylic metallation of a diaryl ether α to a carbamate.

In order to enhance the ease of metallation of the substrates (**6** gives low yield at −78 °C unless sparteine is present), two diols **8** and **9**, available from previous work, were converted to the biscarbamates **10** and **11**. The metallation of *O*-benzylcarbamates has been studied extensively by Hoppe [1,24–26], and the deprotonation of **10** was achieved with *sec*-BuLi in ether and the addition of acetone, returning **12** as a single diastereoisomer (by NMR). Presumably in this case the diastereoisomers still interconvert, but the bulk of the new substituent means that one of the two diastereoisomers is significantly more stable than the other [27]. However we were unable to confirm the relative stereochemistry of the major diastereoisomer of the functionalised products (Scheme 3).

**Scheme 4:**
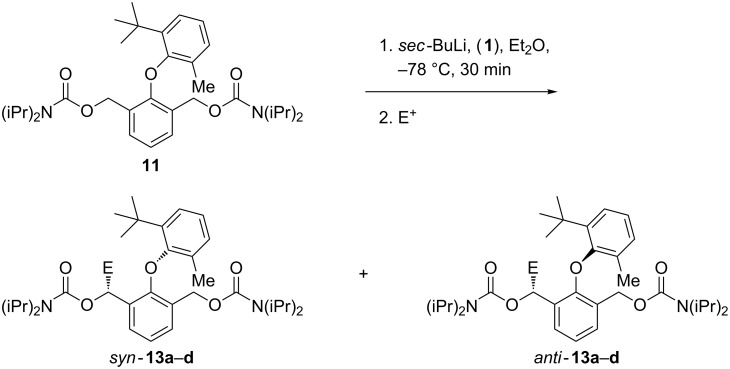
Diastereo- and enantioselective synthesis of atropisomeric ethers by benzylic lithiation.

Moving to the 6’-methyl analogue **11** gave products that were expected to be atropisomeric [10], because they have four substituents ortho to the ether linkage, one of them tertiary. Deprotonation with *sec*-BuLi in Et_2_O at −78 °C and quenching with acetone, cyclobutanone, TMSCl or acetic anhydride all gave good yields of functionalised products with d.r.'s which varied according to the electrophile but were generally high (Scheme 4 and Table 1, entries 1–4). Having established good reactivity in a potentially atropisomeric substrate, we next evaluated the ability of (−)-sparteine [1,3] to control the enantioselectivity of these reactions. Premixing the *sec*-BuLi with (−)-sparteine in Et_2_O, before addition of the reaction substrate and then the electrophile, led to products being formed in high d.r. in each case and with e.r.’s between 73:27 and 81:19 (Table 1, entries 5–8).

**Table 1:** Yields and selectivities in the metallation/quench of **11**.

entry	**1** present?	E^+^ =	E =	product	yield	d.r.	e.r.

1	No	acetone	C(OH)Me_2_	**13a**	75	95:5	–
2	No	cyclobutanone	C(OH)(CH_2_)_2_	**13b**	56	85:15	–
3	No	Me_3_SiCl	SiMe_3_	**13c**	70	95:5	–
4	No	Ac_2_O	COMe	**13d**	69	85:15	–
5	Yes	acetone	C(OH)Me_2_	**13a**	86	>97:3	75:25
6	Yes	cyclobutanone	C(OH)(CH_2_)_2_	**13b**	26	>97:3	73:27
7	Yes	Me_3_SiCl	SiMe_3_	**13c**	72	95:5	78:22
8	Yes	Ac_2_O	COMe	**13d**	66	95:5	81:19

**Scheme 5:**
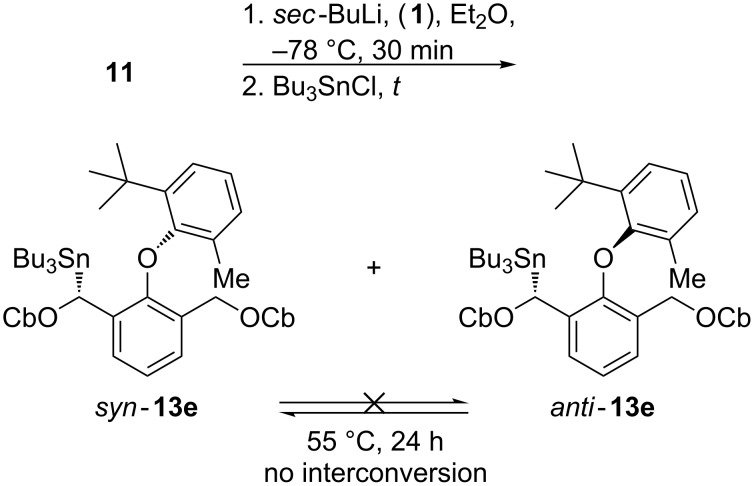
Atroposelective stannylation.

The reaction behaviour was very revealing when the benzyllithiums generated in the presence or absence of (−)-sparteine were quenched with the electrophile Bu_3_SnCl (Scheme 5 and Table 2). Unlike previous examples, the quench provided a product only very slowly; 16 h at −78 °C was required to reach a ca. 60% yield of the stannane **13e** (Table 2, entry 3). After this time the diastereoselectivity was good, but it was clear that this was a result of a slow improvement in the product ratio as the reaction proceeded (Table 2, entries 1–3). Arresting the electrophilic quench after 90 min produced only a 9% yield of a 2:1 ratio of diastereoisomers of **13e**, while intermediate reaction times gave ratios that slowly approached the ratio of 9:1 observed after 16 h. The diastereoisomeric ratios of the products remained unchanged even when the products were heated at 55 °C for 24 h, confirming that the *anti* and *syn* diastereoisomers of the stannanes are stable atropisomers*.*

**Table 2:** Variation of yield and selectivity with quench time.

entry	**1** present?	*t* [h]	yield	d.r.	e.r.

1	No	1.5	9	65:35	–
2	No	6	42	80:20	–
3	No	16	58	90:10	–
4	Yes	16	62	95:5	75:25
5	Yes	26	59	95:5	73:27

This proven conformational stability of the products **13e** indicates that the change in product ratio as the reaction proceeds must be due to a slow change in the structure or diastereoisomeric composition of the lithiated intermediate as the reaction progresses. The results with Bu_3_SnCl suggest that lithiation generates an approximately 20:1 mixture of configurationally stable diastereoisomeric benzyllithiums of which the minor is significantly more reactive than the major (see below). A form of kinetic resolution occurs in which the minor organolithium is rapidly converted to product, followed slowly by the major organolithium [28]. Hoppe observed a related effect in the alkylations of cinnamyllithiums [29].

**Scheme 6:**
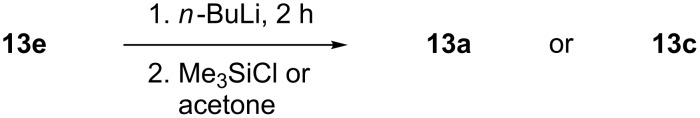
Stereospecific tin–lithium exchange/quench reactions.

Previous results from the laboratories of Hoppe indicated that lithiated *O*-benzylcarbamates are typically configurationally unstable in ether on the macroscopic timescale [1], although Hoffmann detected microscopic configurational stability [30]. To demonstrate that the organolithium intermediates here do have some configurational stability, we treated samples of **13e** of different diastereoisomeric and enantiomeric ratios with *n*-BuLi in ether at −78 °C to induce tin–lithium exchange, quenching the products with either acetone or Me_3_SiCl (Scheme 6).

The major product diastereoisomers were the same as those formed by direct deprotonation quench (Scheme 2). Importantly, in Table 3, entries 1 and 3 show that the diastereoisomeric ratio of the product was at least partly dependent on the d.r. of the starting stannane, necessarily indicating some degree of macroscopic configurational stability. The ratios were not identical, but yields were low, thus differential rates of the reaction of the two diastereoisomeric organolithiums may again be to blame. Enantiomerically enriched stannane returned enantiomerically enriched product, showing that racemisation (which necessarily involves either rotation about the Ar–O–Ar axis or deprotonation–reprotonation) is also slow.

**Table 3:** Stereospecificity in the tin–lithium exchange/quench reactions.

entry	**13e** d.r.	**13e** e.r.	E^+^ =	product, d.r.	e.r.

1	90:10	–	Me_3_SiCl	**13c**, 94:6	–
2	90:10	–	acetone	**13a**, >95:5	–
3	80:20	–	Me_3_SiCl	**13c**, 88:12	–
4	>95:5	80:20	Me_3_SiCl	**13c**, >95:5	80:20

## Discussion

Due to the gummy nature of the products, it turned out to be impossible to establish unequivocally the relative or absolute stereochemistry of the products obtained from the lithiations. However, we can make several important conclusions from this work.

Firstly, racemisation of the intermediate organolithiums is demonstrably slow, because an enantioenriched stannane yields, after tin–lithium exchange and quench, products with conserved e.r. (Table 3, entry 4). The e.r. of the products formed by deprotonation with an alkyllithium-(−)-sparteine complex must therefore be determined during the deprotonation step, in which the alkyllithium-(−)-sparteine complex elects to deprotonate one of the two enantiotopic CH_2_OCb groups of the starting material. Every e.r. produced during this study fell between 75:25 and 81:19, irrespective of the electrophile, so our conclusion is that this selectivity represents the enantioselectivity of the alkyllithium-(−)-sparteine deprotonation step.

Secondly, since epimerisation of the organolithiums is also slow enough for different d.r.'s of a stananne to yield different d.r.'s of a product (Table 3, entries 1 and 3), we can be certain that the secondary benzyllithium centre is macroscopically stable on the timescale of these reactions. This is in contrast with previous reports of benzyllithiums derived from primary benzylcarbamates [1], though not for secondary benzylcarbamates [24], so we assume that steric bulk or electron donation from the ether group is responsible.

Thirdly, the change in ratio of the stannane products in Table 2 with time indicates that two diastereoisomeric organolithiums are formed in unequal quantities. Our best estimate is that the diastereoisomeric ratio of the organolithiums is of the order of 90:10 or 95:5, perhaps better in the presence of (−)-sparteine since d.r.'s were uniformly higher when (−)-sparteine was present (though this may also be due to an improvement in the stereospecificity of the quench).

Fourthly, diastereoselectivity, unlike enantioselectivity, varies significantly according to the electrophile employed, showing that the product d.r. is determined in the electrophilic quench step. Precedent studies suggest that electrophilic quench of benzylcarbamates is typically invertive [24]: All documented stannylations of benzyllithiums are invertive [23], and all documented tin–lithium exchanges (except one, where there is no adjacent heteroatom [31]) are retentive [23]. If these assumptions hold true here, then formation of the same diastereoisomer of the product silane **13a** or alcohol **13c**, by either deprotonation or by stannylation tin–lithium exchange (Table 3, entries 1 and 2), therefore indicates that both of these electrophiles also react with inversion. Lower d.r.'s may result from some electrophiles by competitive reaction with up to 15% retention (Table 1), and the effect of (−)-sparteine on the d.r.'s in Table 1 may be because its steric bulk helps to suppress this competing retentive pathway.

**Scheme 7:**
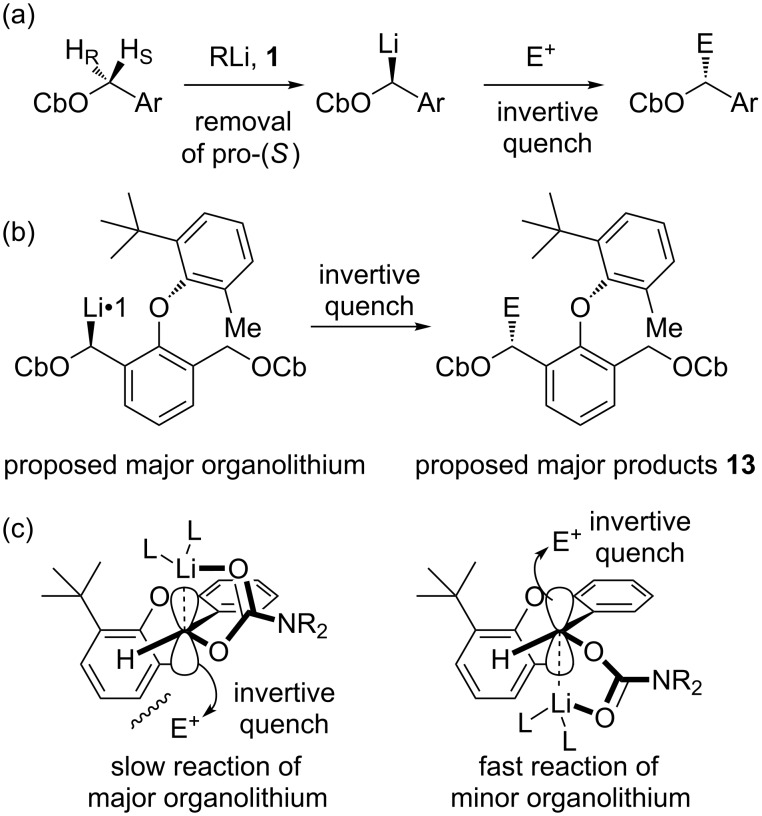
Proposed stereochemical pathway.

Lack of crystallinity has meant that we cannot unequivocally assign absolute or relative stereochemistry in this work. However, a few reasonable assumptions allow us to propose likely assignments, and these are the ones used in the structural diagrams in this paper:

1. (−)-Sparteine favours deprotonation of the pro-*S* proton of carbamates. Invertive quench would result in the formation of the products with stereogenic centres as illustrated in Scheme 7(a).

2. The slower invertive reaction of the major organolithium diastereoisomer with Bu_3_SnCl suggests that the product of this reaction is more hindered (Scheme 7(c)), and we therefore propose that the major diastereoisomers of the reactions are as shown in Scheme 7(b).

## Conclusion

Overall, we have shown in this paper that the use of an alkyllithium-(−)-sparteine deprotonation can desymmetrise a pro-atropisomeric biscarbamoyloxy diarylether, with enantiomeric ratios of up to 81:19. Some mechanistic details of the stereochemical course of the lithiation–substitution reactions have been elucidated, and further work remains to exploit this transformation for the potential synthesis of new classes of chiral ligands [12].

## Supporting Information

File 1 Experimental details and spectral data.
