# Supplementary material for: Carbamate-directed benzylic lithiation for the diastereo- and enantioselective synthesis of diaryl ether atropisomers
Source: Beilstein J Org Chem. 2011 Sep 26;7:1327–33. doi: 10.3762/bjoc.7.156 (PMC3201046; doi:10.3762/bjoc.7.156)

Supporting Information

for

Carbamate-directed benzylic lithiation for the diastereo- and enantioselective synthesis of diaryl ether atropisomers

Abigail Page and Jonathan Clayden*

Address: School of Chemistry, University of Manchester, Oxford Rd., Manchester M13 9PL, UK

Email: Jonathan Clayden - clayden@man.ac.uk

*Corresponding author

Experimental details and spectral data

General procedures:

1. Metallation–quench

The diaryl ether (1 equiv), dissolved in dry Et_2O (10 cm^3), was freeze-thaw degassed under an atmosphere of argon. *sec*-BuLi solution (1.6 equiv) was added dropwise at -78 °C and the reaction mixture was stirred for 30 min (or the specified time if stated). The reaction was quenched with the electrophile and stirred at –78 °C for the time stated. The reaction was quenched by addition of saturated NH_4Cl solution and diluted with EtOAc. The layers were separated and the organic phase was washed with water, brine, dried (Na_2SO_4), and the solvent removed under reduced pressure.

2. Metallation—quench in the presence of (–)-sparteine

Diaryl ether (1 equiv) and freshly distilled (−)-sparteine **1** (1.6 equiv) were dissolved in dry Et_2O and freeze-thaw degassed under an atmosphere of argon. *sec*-BuLi solution (1.6 equiv) was added dropwise at -78 °C and the reaction mixture was stirred for 30 min (or the specified time if stated). The reaction was quenched with the electrophile and stirred at –78 °C for the time stated. The reaction was quenched by addition of saturated NH_4Cl solution and diluted with EtOAc. The layers were separated and the organic phase was washed with water and brine, dried (Na_2SO_4), and the solvent removed under reduced pressure.

3. Tin–lithium exchange–quench

A solution of the stannane (1 equiv) in dry Et_2O (10 cm^3) was freeze-thaw degassed under an atmosphere of Argon. *n*-BuLi solution (1.5 equiv) was added dropwise to the

solution at −78 °C and the reaction mixture was stirred for 2 h (or the specified time if stated). The reaction was quenched with the desired electrophile and stirred at –78 °C with warming to rt over 16 h. The reaction was quenched by addition of saturated NH_4Cl solution and diluted using EtOAc. The layers were separated and the organic phase was washed with water, brine, dried (Na_2SO_4), and the solvent removed under reduced pressure.

2-(2-tert-Butylphenoxy)-1,3-bis(methoxymethyl)benzene (6).

2-(2-*tert*-Butylphenoxy)-1,3-bis(hydroxymethyl)benzene (**8**) (0.4 g, 1.45 mmol, 1 equiv) dissolved in anhydrous THF (10 cm^3) was added under stirring to a suspension of NaH (60% dispersion in mineral oil) (0.35 g, 14.4 mmol, 6 equiv) in THF (10 cm^3) under N_2 and stirred for 30 min at rt. MeI (0.28 cm^3, 4.34 mmol, 3 equiv) was added and the mixture stirred for 16 h, and quenched by addition of water. The solution was diluted using EtOAc, the organic phase washed with water, and brine, dried (Na_2SO_4), and solvent removed under reduced pressure. The crude product was purified by flash column chromatography (2:1 petrol:EtOAc) to yield the product as a white solid (0.39 g, 79%). m.p 103–104 °C, R_f = 0.84 (9:1 petrol:EtOAc). δ_H (300 MHz; $CDCl_3$) 7.50 (2H, d, *J* 7.5, H-1), 7.30-7.26 (2H, m, H-2 and H-3), 7.00-6.95 (2H, m, H-4 and H-5), 6.20 (1H, dd, *J* 8 and 1.5, H-6), 4.4 – 4.2 (4H, CH AB m, H-7), 3.3 (6H, s, H-9) and 1.54 (9H, s, H-8); δ_C (100 MHz; $CDCl_3$) 156.9, 148.8, 136.8, 132.0, 128.6, 127.2, 126.5, 125.6, 121.4, 112.6, 69.3, 58.4, 35.0 and 29.8.; ES+ *m/z* 337 (M + Na); HRMS found M + Na 337.1776, $C_{20}H_{26}O_3Na$ requires 337.1775.

(2-(2-tert-butylphenoxy)-1,3-phenylene)bis(methylene) bis(diisopropylcarbamate) (10).

Diol **8** (0.1 g, 0.35 mmol, 1 equiv), NaH (60% dispersion in mineral oil) (0.08 g, 2.1 mmol, 6 equiv), 18-crown-6 ether (0.002 g), and anhydrous ether (20 cm^3) were charged to a flask at rt and stirred for 30 min. Diisopropylcarbamoyl chloride (0.24 g, 1.40 mmol, 4 equiv) was added to the reaction mixture and the solution heated to reflux (35 °C) for 16 h. The reaction was quenched by addition of H_2O and the organic phase was washed with water and brine, and then dried ($MgSO_4$) and the solvent removed under reduced pressure. The crude product was purified by flash column chromatography (9:1 petrol:EtOAc) to yield the product as a colourless oil turning into a white solid on standing (0.16 g, 87%). m.p. 92–94 °C; R_f = 0.33 (9:1 petrol:EtOAc); δ_H (400 MHz; $CDCl_3$) 7.40 (2H, d, *J* 7.5, H-1), 7.26 (1H, dd, *J* 8 and 2, H-3), 7.22 (1H, t, *J* 7.5, H-2), 6.89-6.79 (2H, m, H-4 and H-5), 6.18 (1H, dd, *J* 8, and 2, H-6), 5.04 – 4.84 (4H AB m, H-8), 3.99 – 3.50 (4H br m, H-9) 1.45 (9H, s, H-7) and 1.15 – 1.00 (24H, br m, H-10); δ_C (100MHz; $CDCl_3$) 156.9, 155.1, 149.9, 137, 131.1, 129.8, 127.3, 127.1, 125.4, 121.6, 112.7, 61.6, 45.9 (br), 35, 29.9, 21.3 and 20.70 (br).; ES+ *m/z* 563.4 (M + Na^+); HRMS found: M + Na^+ 541.3633, $C_{32}H_{48}N_2O_5$ requires 541.3636.

(2-(2-tert-Butyl-6-methylphenoxy)-1,3-phenylene)bis(methylene)bis(diisopropyl-carbamate) (11)

Diol **9** (1.0 g, 3.73 mmol, 1 equiv), NaH (60% dispersion in mineral oil) (0.90 g, 22.35 mmol, 6 equiv), 18-crown-6 ether (0.02 g, *catalytic*), and anhydrous ether (200 cm^3) were charged to a flask at rt and stirred for 30 min. Diisopropylcarbamoyl chloride (1.76 g, 14.9 mmol, 4 equiv) was added to the reaction mixture and the solution

heated to reflux (35 °C) for 16 h. The reaction was quenched by addition of water and the organic phase was washed with water and brine, and then dried ($MgSO_4$) and the solvent removed under reduced pressure. The crude product was purified by flash column chromatography (9:1 petrol:EtOAc) to yield the product as a colourless oil turning into a white solid on standing (1.50 g, 72%). m.p. 96–98 °C. R_f = 0.38 (9:1 petrol:EtOAc); δ_H (400 MHz; $CDCl_3$) 7.26 (2H, d, *J* 7.6, H-1), 7.17 (1H, dd, *J* 7.5 and 1.5, H-3), 7.02 (1H, t, *J* 7.5, H-2), 6.89 (1H, t, *J* 7.5, H-4), 6.84 (1H, dd, *J* 7.5 and 1.5, H-5), 4.96 – 4.82 (4H AB m, H-8), 3.95 – 3.65 (4H br m, H-9) 1.73 (3H, s, H-6), 1.37 (9H, s, H-7) and 1.15 – 1.05 (24H, bd, *J* 6, H-10); δ_C (100MHz; $CDCl_3$) 155.1, 154.5, 152.1, 139.7, 130.5, 129.5, 127.5, 126.9, 125.5, 123.7, 122.6, 61.5, 45.9 (br), 35.3, 30.4, 21.2 (br) and 18.1.; ES+ *m/z* 577 (M + Na^+); HRMS found M + Na 577.3618, $C_{33}H_{50}N_2O_5$ Na requires 577.3612.

Diisopropylcarbamic acid 1-{2-(2-tert-butylphenoxy)-3-[(diisopropylcarbamoyl-oxy)methyl]phenyl}-2-hydroxy-2-methylpropyl ester (12)

Carbamate **10** (0.14 g, 0.25 mmol, 1.0 equiv), *sec*-BuLi (1.3 M in hexanes) (0.31 cm^3, 0.41 mmol, 1.6 equiv), acetone (1 cm^3, excess) and anhydrous ether (14 cm^3) were treated as described in the general procedure. The crude product was purified by flash column chromatography (9:1 petrol:EtOAc) to yield the product as a light yellow oil (0.095 g, 63%). R_f = 0.10 (9:1 petrol:EtOAc); δ_H (400 MHz; $CDCl_3$) 7.55 (1H, dd, *J* 8 and 1.5, H-1), 7.40 (1H, dd, *J* 8 and 1.5, H-1), 7.30-7.20 (2H, m, H-2 and H-3), 6.81 (2H, dd, *J* 6 and 1.5 H-4 and H-5), 6.13 (1H, dd, *J* 6, and 1.5, H-6), 5.80 (1H, br s, H-11), 4.99–4.75 (2H AB m, H-8), 4.00-3.45 (4H, br m, H-9) 1.49 (9H, s, H-7), 1.18 (6H, br s, H-12) and 1.12–1.00 (24H, bm, H-10); δ_C (100MHz; $CDCl_3$) 157.0, 155.4, 155.1, 150.1, 136.8,

132.5, 130.7, 130.0, 128.9, 127.4, 126.9, 125.1, 121.9, 114.0, 76.5, 73.6, 61.9, 45.4 (br), 34.9, 30.1, 27.1, 25.4, and 20.5 (br).; ES+ *m/z* 621.5 (M + Na^+); HRMS found M + H^+ 599.4048, $C_{35}H_{55}N_2O_6$ requires 599.4055.

Diisopropylcarbamic acid 1-{2-(2-tert-butyl-6-methylphenoxy)-3-[(diisopropyl-carbamoyloxy)methyl]phenyl}-2-hydroxy-2-methylpropyl ester (13a)

Carbamate **11** (0.13 g, 0.23 mmol, 1.0 equiv), *sec*-BuLi (1.3 M in hexanes) (0.28 cm^3, 0.37 mmol, 1.6 equiv), acetone (1 cm^3, excess) and anhydrous ether (13 cm^3) were treated as described in general procedure 1. The crude product was purified by flash column chromatography (9:1 petrol:EtOAc) to yield the product as a light yellow oil (0.10 g, 75%).

The product was also synthesised by treatment of carbamate **11** (0.1 g, 0.18 mmol, 1.0 equiv) with *sec*-BuLi (0.22 cm^3, 0.29 mmol, 1.6 equiv), (–)-sparteine (0.07 cm^3, 0. 29 mmol, 1.6 equiv), acetone (1 cm^3, excess) and anhydrous ether (10 cm^3) as described in general procedure 2. The crude product was purified by flash column chromatography (9:1 petrol:EtOAc) to yield the product as a light yellow oil (0.12 g, 86%). HPLC: (R,R-Whelk-O1), eluting with hexane:IPA 97:3 at 1.0 ml/min; retention times 14.04 min (major) and 15.85 min (minor). Material of 50% ee had $[\alpha]_D^{22}$ = +28 (c = 1.000, chloroform).

The product was also synthesised by treatment of stannane **13e** (0.05 g, 0.09 mmol, 1.0 equiv) with *n*-BuLi (2.4 M in hexanes) (0.06 cm^3, 0.14 mmol, 1.5 equiv), acetone (0.5 cm^3, excess) and anhydrous ether (5 cm^3) as described in general procedure 3. The

crude product was purified by flash column chromatography (9:1 petrol:EtOAc) to yield the product as a light yellow oil (0.02 g, 27%).

R_f = 0.13 (9:1 petrol:EtOAc); δ_H (400 MHz; $CDCl_3$) 7.49 (1H, dd, *J* 8 and 1.5, H-1), 7.21-7.15 (2H, m, H-1 and H-3), 7.05 (1H, t, *J* 8, H-4), 6.86 (1H, t, *J* 7.5, H-2), 6.78 (1H, dd, *J* 7.5, and 1, H-5), 6.51 (1H, s, H-11) 4.62 – 4.32 (2H AB m, H-8), 3.93–3.50 (4H br m, H-9), 1.6 (3H, s, H-6), 1.41 (9H, s, H-7), 1.24 (6H, d, *J* 4, H-12), and 1.10 (24H, dd, *J* 21 and 7, H-10); δ_C (100MHz; $CDCl_3$) 155.2, 155.0, 152.4, 139.3, 130.9, 130.2, 129.7, 129.1, 127.8, 126.4, 125.4, 123.6, 122.5, 75.5, 73.8, 60.8, 46.0 (br), 35.4, 30.3, 26.9, 26.0, 21.4 (br) and 18.2.; ES+ *m/z* 635 (M + Na^+), HRMS found M + H^+ 613.4229, $C_{36}H_{57}N_2O_6$ requires 613.4212.

Diisopropylcarbamic acid {2-(2-tert-butyl-6-methyl-phenoxy)-3-[(diisopropyl-carbamoyloxy)methyl]phenyl}-(1-hydroxycyclobutyl)methyl ester (13b)

Carbamate **11** (0.1 g, 0.18 mmol, 1.0 equiv), *sec*-BuLi (1.3 M in hexanes) (0.22 cm^3, 0.29 mmol, 1.6 equiv), cyclobutanone (1 cm^3, excess) and anhydrous ether (10 cm^3) were treated as described in general procedure 1. The crude product was purified by flash column chromatography (9:1 petrol:EtOAc) to yield the product as a clear gum (0.06 g, 56%).

The product was also synthesised by treatment of carbamate **11** (0.1 g, 0.18 mmol, 1.0 equiv) with *sec*-BuLi (0.22 cm^3, 0.29 mmol, 1.6 equiv), (–)-sparteine (0.07 cm^3, 0. 29mmol, 1.6 equiv), cyclobutanone (1 cm^3, excess) and anhydrous ether (10 cm^3) as described in general procedure 2. The crude product was purified by flash column chromatography (9:1 petrol:EtOAc) to yield the product as a clear gum (0.028 g, 26%).

HPLC: (R,R-Whelk-O1), eluting with hexane:IPA 97:3 at 1.0 ml/min; retention times 14.79 min (major) and 16.89 min (minor). Material of 46% ee had $[\alpha]_D^{22}$ = +9 (c = 0.100, chloroform).

*R*f = 0.10 (9:1 petrol:EtOAc); δ_H (500 MHz; $CDCl_3$) 7.66 (1H dd, *J* 7.5 and 1.5, H-1), 7.30 (1H, dd, *J* 8 and 1, H-1), 7.24 (1H, br d, *J* 7, H-3), 7.15 (1H, t, *J* 7.5, H-2), 6.94 (1H, t, *J* 7.5, H-4), 6.87 (1H, br d, *J* 7, H-5), 6.67 (1H, br s, H-11), 4.73-4.45 (2H, AB m, H-8), 4.05-3.60 (4H, br m, H-9), 3.22 (1H, s, H-14), 2.55-2.50 (1H, br m, H-12), 2.35-2.28 (1H, br m, H-12), 2.17-1.95 (4H, br m, H-12 and H-13), 1.70 (3H, s, H-6), 1.26 (9H, s, H-7), 1.22-1.15 (24 H, br m, H-10).; δ_C (100MHz; $CDCl_3$) 155.1, 155.0, 152.2, 139.3, 130.95, 130.0, 129.0, 127.8, 126.5, 125.4, 123.6, 122.6, 78.0, 73.9, 60.8, 45.9 (br), 35.4, 33.4, 320, 30.4, 21.1 (br), 18.3 and 12.5.; ES+ *m/z* 647 (M + Na^+); HRMS found M + Na 647.4028, $C_{37}H_{56}N_2O_6$ Na requires 647.4036.

Diisopropylcarbamic acid {2-(2-tert-butyl-6-methylphenoxy)-3-[(diisopropyl-carbamoyloxy)methyl]phenyl}trimethylsilanylmethyl ester (13c)

Carbamate **11** (0.1 g, 0.18 mmol, 1.0 equiv), *sec*-BuLi (1.3 M in hexanes) (0.22 cm^3, 0.29 mmol, 1.6 equiv), TMSCl (1 cm^3, excess) and anhydrous ether (10 cm^3) were treated as described in general procedure 1. The crude product was purified by flash column chromatography (9:1 petrol:EtOAc) to yield the product as a light yellow oil (0.079 g, 70%).

The product was also synthesised by treatment of carbamate **11** (0.1 g, 0.18 mmol, 1.0 equiv) with *sec*-BuLi (0.22 cm^3, 0.29 mmol, 1.6 equiv), (–)-sparteine (0.07 cm^3, 0. 29 mmol, 1.6 equiv), TMSCl (1 cm^3, excess) and anhydrous ether (10 cm^3) as described in general procedure 2. The crude product was purified by flash column chromatography

(9:1 petrol:EtOAc) to yield the product as a light yellow oil (0.08 g, 72%). HPLC: (R,R-Whelk-O1), eluting with hexane:IPA 97:3 at 1.0 ml/min; retention times 9.54 min (major) and 10.81 min (minor). Material of 56% ee had $[\alpha]_D^{22}$ = +52 (c = 1.000, chloroform).

The product was also synthesised by treatment of stannane **13e** (0.05 g, 0.09 mmol, 1.0 equiv) with *n*-BuLi (2.4 M in hexanes) (0.06 cm^3, 0.14 mmol, 1.5 equiv), TMSCl (0.5 cm^3, excess) and anhydrous ether (5 cm^3) as described in general procedure 3. The crude product was purified by flash column chromatography (9:1 petrol:EtOAc) to yield the product as a light yellow oil (0.037 g, 33%).

R_f = 0.60 (9:1 petrol:EtOAc); δ_H (400 MHz; $CDCl_3$) 7.31 (1H, dd, *J* 8 and 2, H-1), 7.20 (1H, dd, *J* 8 and 2, H-1), 7.16 (1H, dd, *J* 8 and 2, H-3), 7.08 (1H, t, *J* 8, H-2 or H-4), 6.90 (1H, t, *J* 8, H-2 or H-4), 6.84 (1H, br dd, *J* 7.5 and 1, H-5), 6.28 (1H, s, H-11), 4.63-4.35 (2H, AB m, H-8), 3.90-3.76 (4H, br m, H-9), 1.64 (3H, s, H-6), 1.36 (9H, s, H-7) 1.11 (12H, br d, *J* 4, H-10), 1.04 (12H, br d, *J* 4, H-10) and 0.00 (9H, s, H-12).; δ_C (100MHz; $CDCl_3$) 153.7, 153.5, 149.1, 137.3, 131.5, 129.6, 126.7, 126.3, 125.0, 123.6, 121.6, 121.2, 64.3, 59.5, 44.6 (br), 33.9, 28.8, 19.7 (br), 16.8 and -4.2.; ES+ *m/z* 649 (M + Na^+), HMRS Found M + Na 649.3996 $C_{36}H_{58}N_2O_5Si$ Na requires 649.4008.

Diisopropylcarbamic acid 1-{2-(2-tert-butyl-6-methylphenoxy)-3-[(diisopropyl-carbamoyloxy)methyl]phenyl}-2-oxopropyl ester (13d)

Carbamate 11 (0.1 g, 0.18 mmol, 1.0 equiv), *sec*-BuLi (1.3 M in hexanes) (0.22 cm^3, 0.29 mmol, 1.6 equiv), acetic anhydride (1 cm^3, excess) and anhydrous ether (10 cm^3) were treated as described in general procedure 1. The crude product was purified by flash column chromatography (9:1 petrol:EtOAc) to yield the product as a light yellow oil (0.11 g, 69%).

The product was also synthesised by treatment of carbamate **11** (0.1 g, 0.18 mmol, 1.0 equiv) with *sec*-BuLi (0.22 cm^3, 0.29 mmol, 1.6 equiv), (–)-sparteine (0.07 cm^3, 0. 29mmol, 1.6 equiv) and acetic anhydride (1.0 cm^3) as described in general procedure 2. The crude product was purified by flash column chromatography (9:1 petrol:EtOAc) to yield the product as a light yellow oil (0.10 g, 66%). HPLC: (R,R-Whelk-O1), eluting with hexane:IPA 97:3 at 1.0 ml/min; retention times 11.89 min (major) and 16.11 min (minor). Material of 60% ee had $[\alpha]_D^{22}$ = +102 (c = 0.9, chloroform).

R_f = 0.29 (9:1 petrol:EtOAc); δ_H (400 MHz; $CDCl_3$) 7.38 (1H, dd, *J* 7.5 and 2, H-1), 7.33-7.29 (2H, m, H-1 and H-3), 7.13 (1H, t, *J* 7.5, H-2), 6.98 (1H, t, *J* 8, H-4), 6.88 (1H, dd, *J* 7.5 and 1, H-5), 6.54 (1H, s, H-11), 4.92-4.70 (2H, AB m, H-8), 4.05-3.75 (4H, br m, H-9), 2.14 (3H, s, H-12), 1.74 (3H, s, H-6), 1.52 (9H, s, H-7), 1.35-1.15 (24H, br m, H-10).; δ_C (100MHz; $CDCl_3$) 202.8, 154.5, 154.3, 152.7, 139.4, 131.2, 130.7, 129.6, 127.6, 127.4, 125.7, 124.0, 123.3, 74.1, 61.1, 45.6 (br), 35.4, 30.5, 26.8, 20.9 (br), and 18.3.; ES+ *m/z* 619 (M + Na^+), HRMS Found M + H 597.3889, $C_{35}H_{53}N_2O_6$ requires 597.3899.

Diisopropylcarbamic acid {2-(2-tert-butyl-6-methyl-phenoxy)-3-[(diisopropyl-carbamoyloxy)methyl]phenyl}tributylstannanylmethyl ester (13e)

Carbamate **11** (0.1 g, 0.18 mmol, 1.0 equiv), *sec*-BuLi (1.3 M in hexanes) (0.22 cm^3, 0.29 mmol, 1.6 equiv), $SnBu_3Cl$ (0.5 cm^3, excess) and anhydrous ether (10 cm^3) were treated as described in general procedure 1. The crude product was purified by flash column chromatography (9:1 petrol:EtOAc) to yield the product as a light yellow oil (0.088 g, 58%) and as a mixture of diastereomers in the ratio 9:1. HPLC: minor diastereomer elutes first, (R,R-Whelk-O1), eluting with hexane:IPA 97:3 at 1.0 ml/min;

retention times 3.13 min and 6.29 min; major diastereomer elutes second, (R,R-Whelk-O1), eluting with hexane:IPA 97:3 at 1.0 ml/min; retention times 5.84 min and 6.63 min; The product was also synthesised by treatment of carbamate **11** (0.1 g, 0.18 mmol, 1.0 equiv) with *sec*-BuLi (0.22 cm^3, 0.29 mmol, 1.6 equiv), (–)-sparteine (0.07 cm^3, 0. 29mmol, 1.6 equiv), Bu_3SnCl (1 cm^3, excess) and anhydrous ether (10 cm^3) as described in general procedure 2. The crude product was purified by flash column chromatography (9:1 petrol:EtOAc) to yield the product as a light yellow oil (0.094 g, 62%). HPLC: (R,R-Whelk-O1), eluting with hexane:IPA 97:3 at 1.0 ml/min; retention times 5.84 min (minor) and 6.63 min (minor). Material of 50% ee had $[\alpha]_D^{22}$ = –21 (c = 1.000, chloroform).

R_f = 0.29 (9:1 petrol:EtOAc); δ_H (400 MHz; $CDCl_3$) 7.27-7.16 (3H, m, H-1 and H-3), 7.07 (1H, t, *J* 7.5, H-2 or H-4), 6.97 (1H, t, *J* 7.5, H-2 or H-4), 6.91 (1H, dd, *J* 7.5 and 1.5, H-5), 5.92 (1H, s, H-11_{MINOR}), 5.50 (1H, s, H-11_{MAJOR}), 5.10-4.98 (2H, AB m, H-8_{MAJOR}), 4.75-4.55 (2H, s, H-8_{MINOR}), 4.10-3.85 (3H, br m, H-9), 3.70 – 3.60 (1H, br m, H-9), 1.80 (3H, s, H-6), 1.41 (9H, s, H-7), 1.40-1.27 (9H, m, H-14), 1.22-1.14 (24H, br m, H-10), 1.02 (3H, br m, H-12), 0.84 (9H, t, *J* 7.5, H-12), 0.72 (6H, q, *J* 8, H-13).; δ_C (100MHz; $CDCl_3$) 155.4, 155.2, 151.2, 139.4, 130.6, 130.5, 127.3, 126.8, 125.7, 123.6, 122.5, 123.3, 74.1, 62.0, 58.08, 46.1 (br), 35.4, 30.4, 29.1, 27.6, 21.5 (br), 20.5, 18.6, 13.7 and 10.7.; ES+ *m/z* 867 (M + Na^+).

Representative NMR Spectra

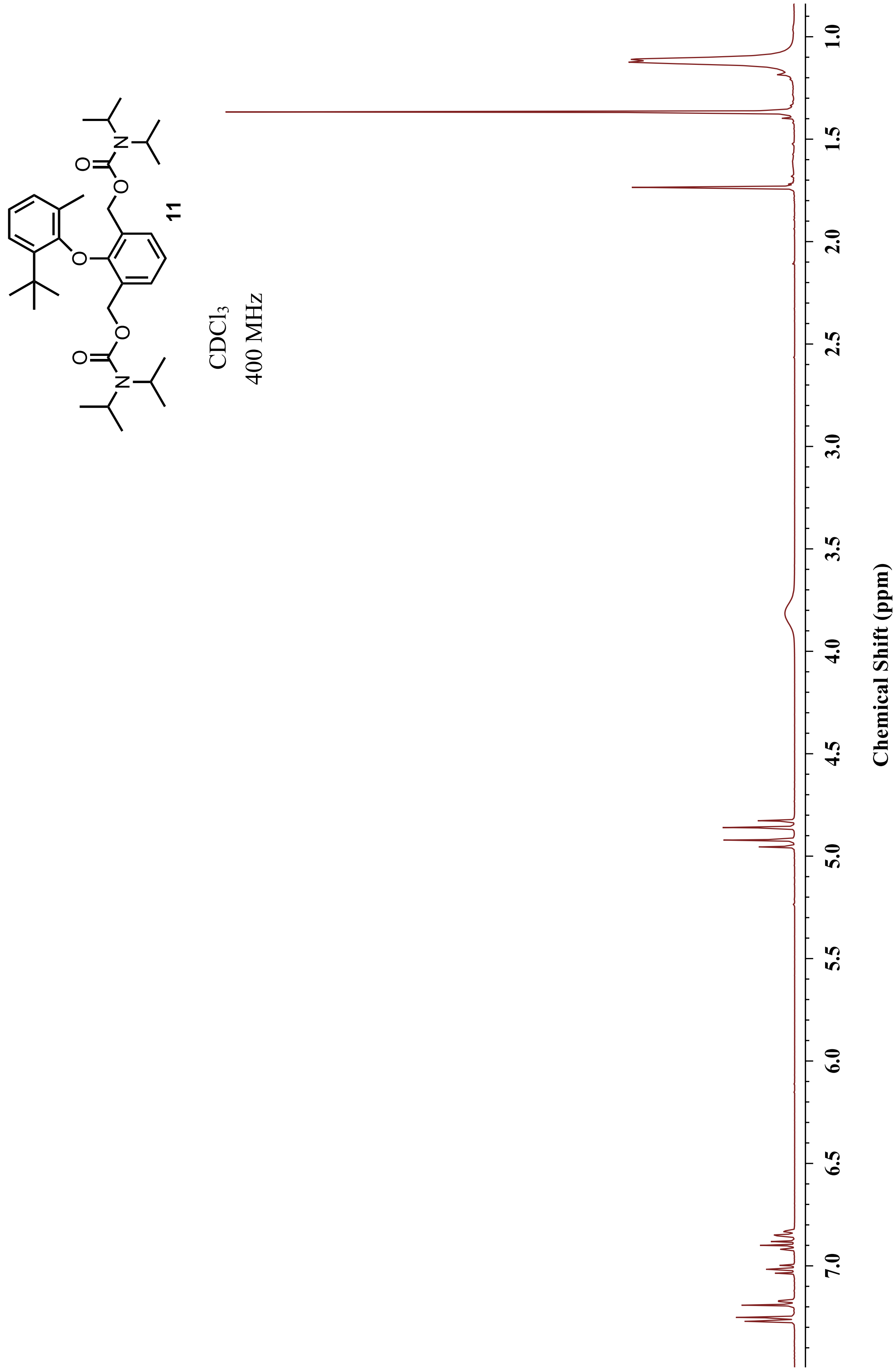

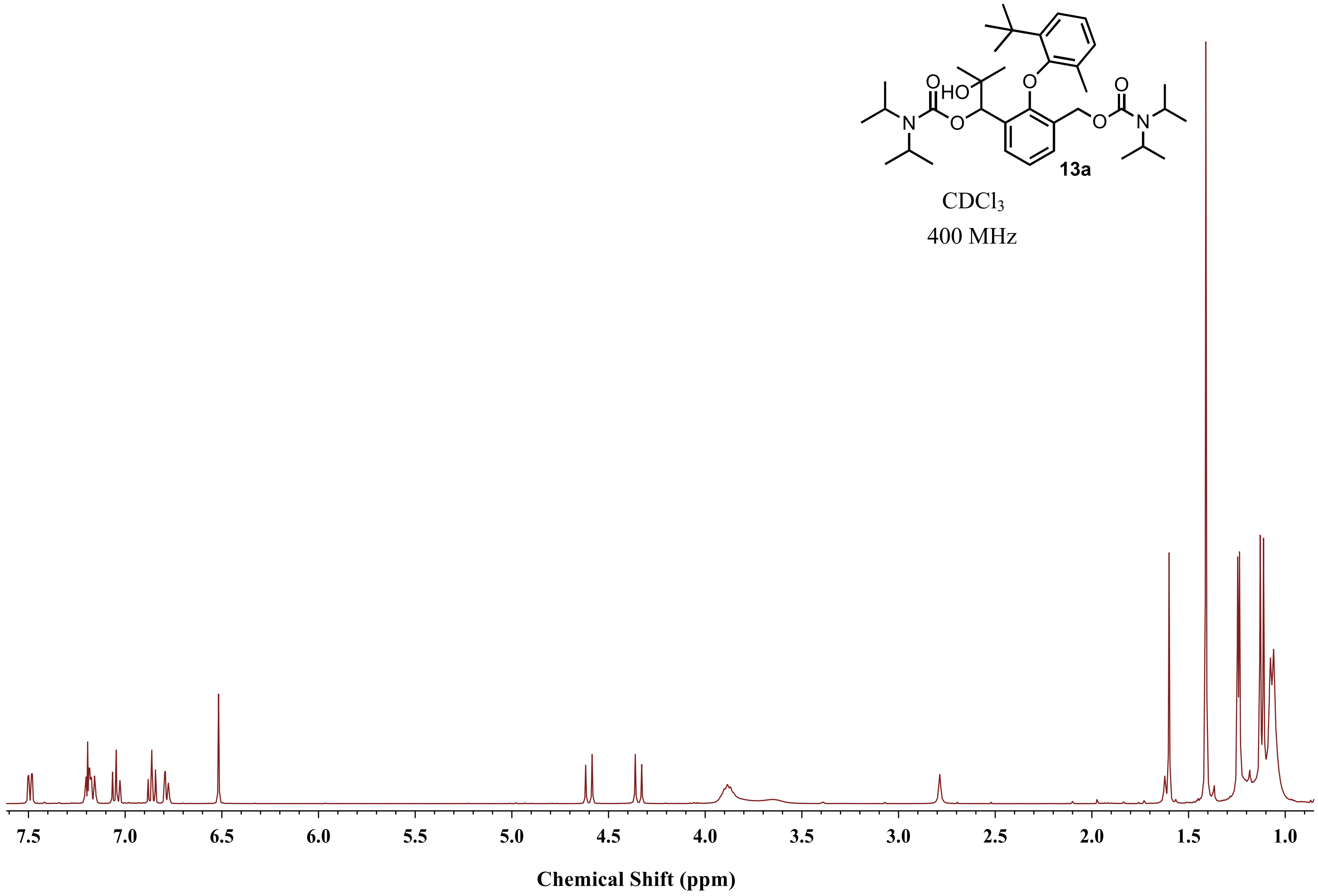
13a
$CDCl_3$
400 MHz
7.5 7.0 6.5 6.0 5.5 5.0 4.5 4.0 3.5 3.0 2.5 2.0 1.5 1.0
Chemical Shift (ppm)

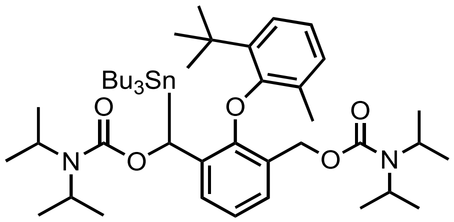

13e
2:1 ratio of diastereoisomers

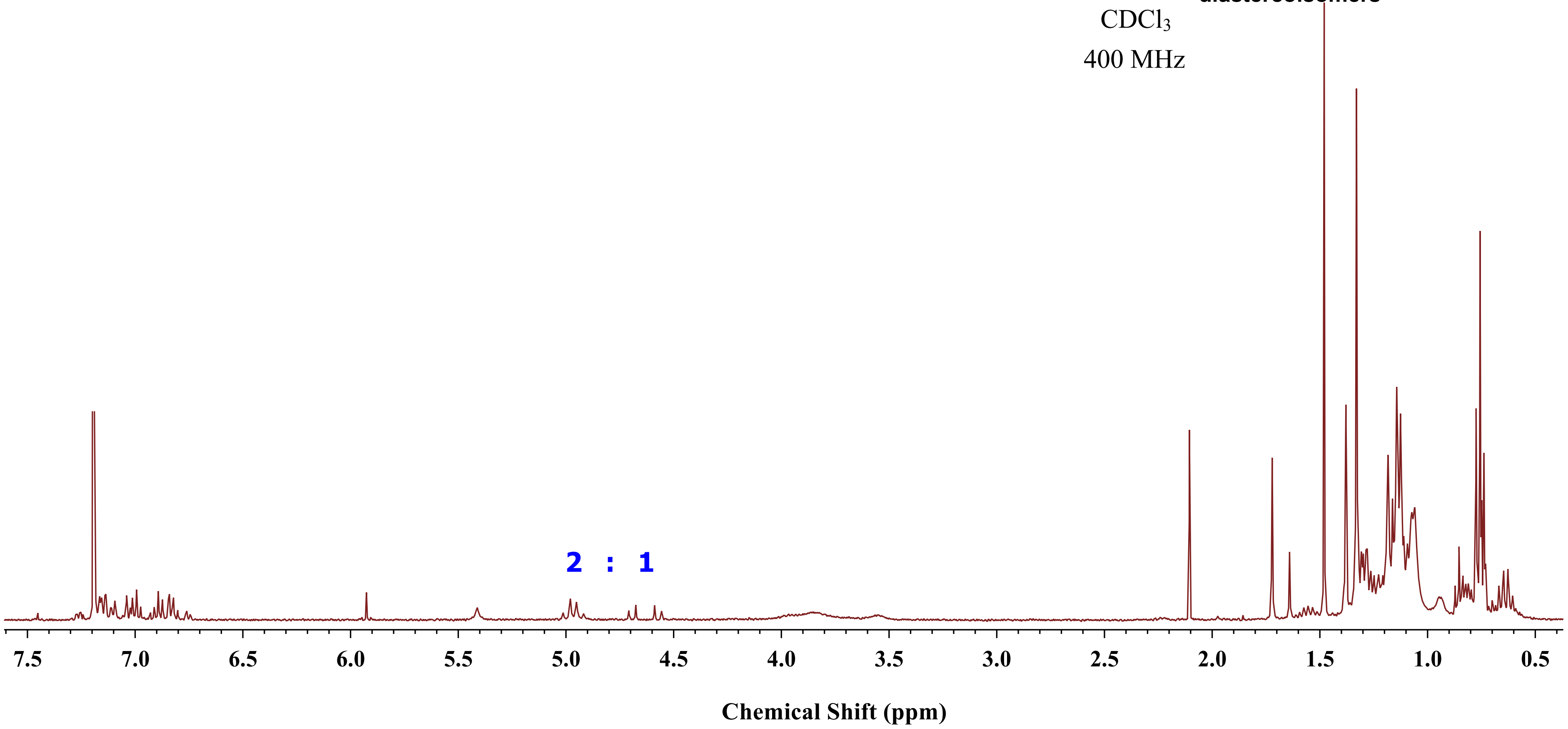

Supplement: File 1 — Experimental details and spectral data. [file Beilstein_J_Org_Chem-07-1327-s001.pdf]
